# Evidence of genotypic adaptation to the exposure to volcanic risk at the dopamine receptor *DRD4* locus

**DOI:** 10.1038/srep37745

**Published:** 2016-12-01

**Authors:** Charlotte Faurie, Clement Mettling, Mohamed Ali Bchir, Danang Sri Hadmoko, Carine Heitz, Evi Dwi Lestari, Michel Raymond, Marc Willinger

**Affiliations:** 1 Institute of Evolutionary Sciences (ISEM), University of Montpellier, 2 Place Eugène Bataillon, 34090 Montpellier, France; 2 Institute of Human Genetics, UPR 1142 CNRS, 141 rue de la Cardonille 34396 Montpellier, France; 3 Ecole Nationale du Génie de l’Eau et de l’Environnement, 1 Quai Koch, 67070 Strasbourg, France; 4 Department of Geography and Environmental Science, Faculty of Geography, Universitas Gadjah Mada, Yogyakarta, Indonesia; 5 Laboratoire Montpelliérain d’Economie Théorique et Appliquée, University of Montpellier, Bâtiment 26, 2 Place Pierre Viala, 34060 Montpellier, France

## Abstract

Humans have colonized and adapted to extremely diverse environments, and the genetic basis of some such adaptations, for example to high altitude, is understood. In some cases, local or regional variation in selection pressure could also cause behavioural adaptations. Numerous genes influence behaviour, such as alleles at the dopamine receptor locus D4 (*DRD4*), which are associated with attitude toward risk in experimental settings. We demonstrate genetic differentiation for this gene, but not for five unlinked microsatellite loci, between high- and low risk environments around Mount Merapi, an active volcano in Java, Indonesia. Using a behavioural experiment, we further show that people inhabiting the high risk environment are significantly more risk averse. We provide evidence of a genetic basis for this difference, showing that heterozygotes at the *DRD4* locus are more risk averse than either homozygotes. In the high risk environment, allele frequencies are equilibrated, generating a high frequency of heterozygotes. Thus it appears that overdominance (i.e. selective advantage of heterozygotes) generates negative frequency dependent selection, favouring the rarer allele at this locus. Our results therefore provide evidence for adaptation to a marginal habitat through the selection of a neurocognitive trait with a genetic basis.

Humans occupy highly heterogeneous environments and this environmental heterogeneity is likely to have selected for locally adapted traits related to personality and habitat preferences. Hundreds of millions of people around the world occupy habitats at volcanic risk. For such environments with a very high level of background risk (an unavoidable and uninsurable independent risk), specific behavioural traits for the ability to cope with such environments may be selected, potentially generating local adaptation in these traits. Adaptation to a marginal habitat may occur if three conditions are met: 1) the average migration distance is smaller than the size of the risky environment (so that offspring produced in the risky environment most likely remain in that environment), 2) the behavioural traits that increase the ability to cope with the risky environment are heritable, and 3) there is a benefit (counterbalancing vital risks) to live in the risky area^1^,^2^.

Genes involved in the regulation of the dopaminergic system are good candidates. Dopamine is a neurotransmitter related to the pleasure system in the brain that provides reinforcement for behaviours associated with the expectation of reward. The dopamine receptor gene D4 (*DRD4*) is expressed in the prefrontal cortex, showing an unusually variable repeat region in the third cytoplasmic loop, which codes for 16 amino acids with between 2 and 11 repeats (2 R to 11 R), making it highly polymorphic^3^,^4^. The 4 R variant is the ancestral, and most common, allele in all human populations^5^. The *DRD4* locus is known to be under selection^6^ and molecular evidence for selection has been uncovered^7^,^8^. The 7 R allele has been linked to novelty-seeking^9^,^10^ although this is controversial^11^. Moreover, it has been found to be associated with financial risk-taking^12^,^13^,^14^ and accounts for 20% of heritable variation in this trait.

## Results

Mount Merapi (Java, Indonesia) is a long-active volcano, with dangerous and often deadly pyroclastic flows, about 70 since the first recorded eruption in 1548, every 2,5 ± 2,1 years (mean ± SD) since the 19^th^ century^15^. Due to the shape of the volcano, the dangerous flows only hurtle down towards the South or South-West, and the North slope is safe. However, living in the risky area allows a third harvest per year and lucrative mineral extraction, providing the incentive for inhabiting this risky and unpredictable environment. Population samples from risky areas (4 villages, *N* = 94) and non-risky areas (7 villages, *N* = 164) were compared. On average, the distance from birthplace to place of residence was 1.7 ± 0.1 km (estimation based on 2,808 individuals, including participants, their parents, and grandparents), which is small compared to the size of the risky area (500–800 km^2^), suggesting that only moderate selective effects could generate local adaptation. Moreover, the average migration distance recently increased: it was almost 6 times lower just two generations ago (4.82 km for the participants in our study vs. 0.81 km for their grandparents, Wilcoxon *P* < 10^−15^). The risky and non-risky areas did not significantly differ on socio-demographic variables such as sex ratio (Fisher exact test *P* = 0.4), age (Wilcoxon *P* = 0.5), education level (Fisher’s exact test *P* = 0.1), and income (Wilcoxon *p* = 0.4), or on physical variables such as body mass index (Wilcoxon *P* = 0.4). However, the individual perception of volcanic risk and the number of volcanic eruptions experienced in the last 10 years were higher in the risky area (Wilcoxon *P* < 0.001): the inhabitants’ subjective perception of background risk is therefore in agreement with objective data on volcanic activity.

**Figure 1:**
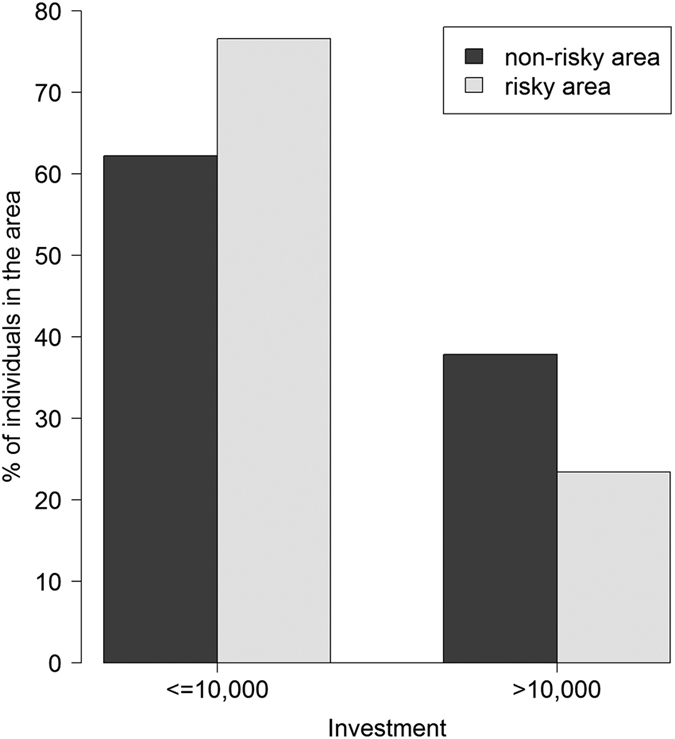
Proportion of individuals investing less versus more than 10,000 IDR in the portfolio choice task. Individuals could choose to invest any amount between 0 and 20,000 in the risky option, by units of 500 IDR. Individuals in the risky area (in grey) tend to be more risk-averse i.e. to invest less (on average 10,723 ± 529 SEM) than in the non-risky area (in black, 12,241 ± 450).

Our experimental measurement of risk-taking, which is based on a simple portfolio choice task^16^ showed that risk-tolerance differed between high and low risk environments. People living in the risky environment chose to invest less money in the risky option (Fig. 1), indicating that they are significantly more risk averse (Wilcoxon test, *W* = 8838, *P* = 0.038). This was also significant in a censored regression model analysing the effect of resident environment on investment (X^2^ = 4.3, df = 1, *P* = 0.038) while controlling for sex (women invest less, *P* < 0.001) and income (rich people invest more, *P* < 0.001), as is generally found in such studies^17^. The effect of age was not significant (*P* = 0.3). Controlling for education instead of income provided similar results. The difference was especially striking for the highest investors (those who invested their whole endowment in the risky option): there were many fewer such individuals in the risky environment (Fig. S1). Our field data are consistent with both theoretical predictions^18^,^19^ and data from laboratory experiments^20^,^21^,^22^,^23^,^24^ showing that individuals who are exposed to substantial background risk are less willing to invest in a risky option in accordance with the “risk-vulnerability hypothesis”^18^.

Genotypes at the *DRD4* locus displayed two common alleles (4 R and 2 R, with 4 and 2 repeats, respectively), as expected in a South-East Asian population^5^,^25^, and 4 minor alleles of negligible frequencies (3 R, 5 R, 6 R, and 7 R), leading to 3 common genotypes (2 R/2 R, 2 R/4 R and 4 R/4 R) and 7 uncommon genotypes (Table 1). Within each area, Hardy-Weinberg equilibrium was not rejected (Table 1). Between the risky and non-risky areas, there was a significant differentiation (exact test, *P* = 0.010; F_ST_ = 0.023) on *DRD4* genotypes (Table 2). This differentiation was due to the most prevalent alleles: 4 R was less common in the risky area (48%) than in the non-risky area (59%), and the opposite was true for allele 2 R (48% and 36% respectively). Considering that this genetic differentiation took place within a geographic range of less than 90 km (the largest distance between samples), a distance for which substantial genetic differentiation is not usually reported^26^,^27^, this suggests that differential selection on *DRD4* alleles in the two areas has been relatively high. This degree of genetic differentiation could reflect recent contact between distinct populations but our data for five unlinked microsatellite loci reject such an explanation. We found low (F_ST_ = −0.0008) genotypic differentiation at these five loci between the risky and the non-risky areas that was not significantly different from zero (*P* = 0.25, Table 2), suggesting that the genetic differentiation observed at *DRD4* is not due to a phenomenon affecting the entire genome, such as admixture between two populations or different mean relatedness between the two areas. In addition, the mean migration pattern does not differ between the two areas (Wilcoxon *P* = 0.9, N = 459 participants and their spouses), suggesting that relatedness does not vary between the two areas.

**Figure 2:**
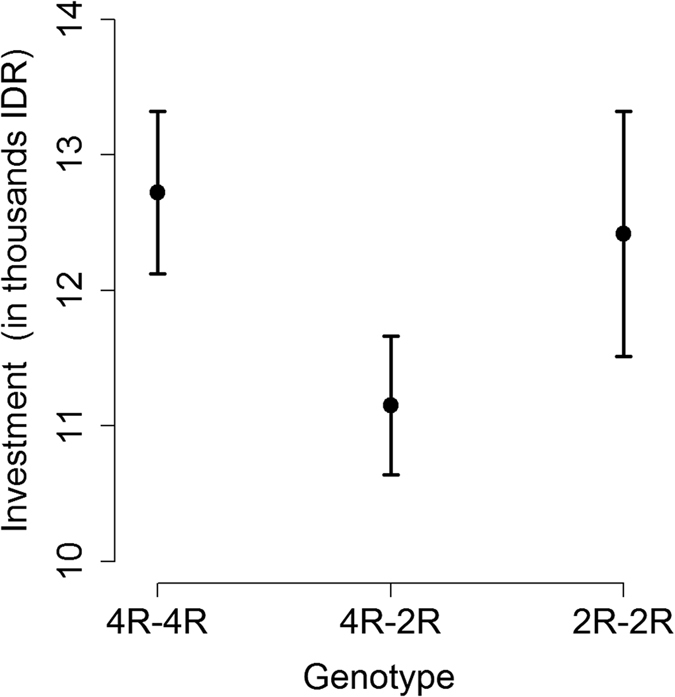
Average investment (in IDR) in the risky option, as a function of an individual’s genotype. Heterozygotes 4R-2R tend to be more risk-averse, i.e. to invest less money (11,150 ± 512 SEM) than homozygotes 4R-4R or 2R-2R (12,724 ± 600 or 12,417 ± 905, respectively).

The amount of money that our respondents invested in the risky option was not independent of their genotype at the *DRD4* locus (Fig. 2, Fig. S2). To investigate whether *DRD4* genotype influenced investment choice, we added genotype (4R-4R, 4R-2R, 2R-2R) to the previous censored regression model (Table 3, Table S1). We found that 4R-2R heterozygotes were more risk averse than either 4R-4R or 2R-2R homozygotes (*P* = 0.005, with 2.3% of the total variance in investment explained by this variable, to be compared to 4.0% and 3.1% of the variance explained by sex and income, respectively), regardless of whether they lived in a high or low risk environment. In fact, after adding genotype to the model, the effect of home environment was no longer significant (*P* = 0.13), and no significant interaction was found between home environment and genotype (*P* = 0.25), providing no evidence for behavioural plasticity of an individual, with a given genotype, following exposure to high background risk. Therefore, although these findings are consistent with the risk vulnerability hypothesis at the population level, with high risk populations showing higher mean risk-aversion, the risk vulnerability hypothesis is not supported at the individual level. The behavioural differences we observed between risky and non-risky environments (Fig. 1) could be explained by an effect of genotype on behaviour, combined with the distribution of genotypes across environments.

**Figure 3:**
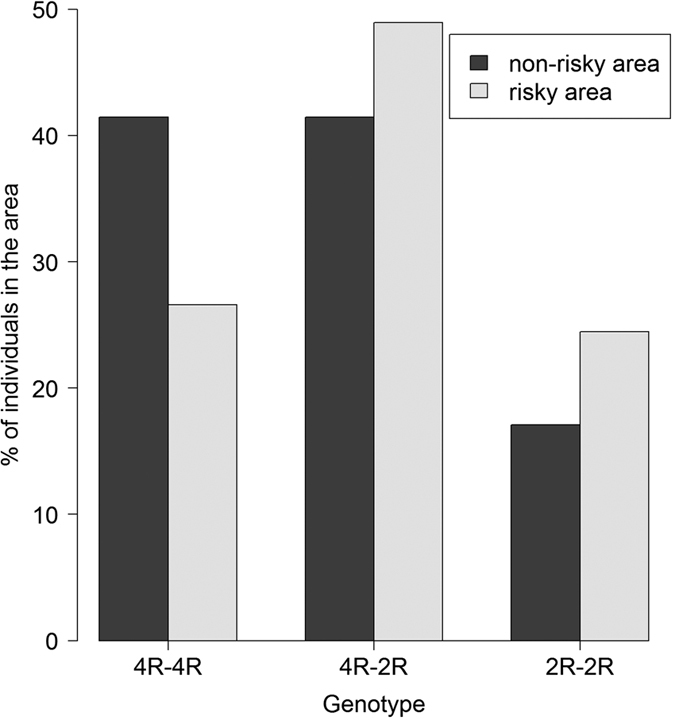
Proportion of individuals with the three possible genotypes in the risky area (in grey) and in the non-risky area (in black). Heterozygotes 4R-2R in the risky area are almost at the maximum possible frequency (50%) for a randomly mating population. Sample sizes: 93 individuals with the genotype 4R-4R, 114 with 4R-2R, and 51 with 2R-2R.

As was already suspected^7^,^8^, this is a case of overdominance, with 2R-2R and 4R-4R homozygotes sharing a similar phenotype (see contrasts in Table 3: P = 0.9). Correspondingly, we found that 4R-2R heterozygotes were more common in the high- than in the low risk environment (Fisher’s exact test: P = 0.01; Fig. 3, Fig. S3), because frequencies of the two common alleles are more equilibrated. Thus it appears that overdominance generates negative frequency-dependent selection favouring the rarer allele in high risk environments to produce the highest frequency of heterozygous, risk-averse, individuals. Our findings therefore indicate that exposure of a population to a risky environment does not induce evolution toward risk tolerance but instead toward risk aversion, as an evolutionary extension of the risk vulnerability hypothesis. An explanation could be that risk-averse individuals have higher reproduction or survival when exposed to fatal risk. The mechanisms involved, allowing better survival chances for individuals and their offspring (e.g. a safer behaviour during volcanic eruptions or a better preparation to such events), remain to be investigated. Our results could alternatively have been consistent with differential migration between phenotypes, however, migration distances do not differ between genotypes (Kruskal-Wallis 0.31142, p = 0.86) or between phenotypes (Wilcoxon W = 5100.5, p = 0.61). Differential selection is therefore the most likely explanation.

## Discussion

There is a balanced selection acting on *DRD4* (as already suspected^7^), through the mechanism of overdominance. The molecular mechanism underlying this advantage of heterozygotes in the risky area remains unknown. *DRD4*, like the D2 dopamine receptor family to which it belongs and many other G-protein-coupled receptors, dimerises to transduce a signal^28^. Selection could be due to differences between the activity of heterodimers and homodimers. Interestingly, the effect of the 7 R allele on risk-taking behaviour previously reported by Dreber *et al*.^12^ may also be an effect of the heterozygote (in that case 4R-7R). Indeed, 7 R is a minor allele in the Western population studied, the frequency of 7 R homozygotes being less than 2%, so the effect of the 7 R allele in the studied population was mainly observed in heterozygotes and a far larger sample would have been required to detect an effect of homozygotes for this allele. Interestingly, the fact that a significant frequency difference was reached with a relatively modest sample size (N = 258) suggests that such selection on *DRD4* alleles could be easily investigated in other volcanic areas or other risky environments. Altogether, these results provide evidence for human genetic local adaptation to a specific environment, and contribute to elucidating the links between genes and the preferences involved in risk-taking attitudes and behaviours.

## Methods

A field study was conducted at Mount Merapi, Java, Indonesia, between November 2012 and August 2013. All experiments were performed in accordance with relevant guidelines and regulations. The protocol (including DNA genotyping) was approved by the Indonesian Ministry of Research and Technology and informed consent was obtained from all participants. During the behavioural measures, genotypes of the individuals were not yet established. The investigators were blind to the behavioural measures during the genotyping.

### Measurement of risk tolerance

We relied on a standard technique (portfolio choice task) to elicit respondents risk tolerance^16^. The experimental instructions for participants were translated from French into Bahasa Indonesia and back-translated for consistency check. Each respondent was given an amount of cash of 20,000 IDR. They were invited to choose the portion of this amount (between 0 and 20,000, by units of 500 IDR) that they wished to invest in a risky option. The rest of the money was accumulated on their total balance.

The risky investment was explained as follows: *There is an equal chance that the investment will fail or succeed. If the investment fails, you lose the amount you invested. If the investment succeeds, you receive 3 times the amount invested. How do we determine if you win or lose? After you have chosen how much to invest, a young child’s “innocent hand” will choose a ball in the urn. If the ball is blue you win, if it is red you lose.*

Three examples were provided in the instructions:

Example 1: *If you choose to invest nothing, you will get the 20,000 Rupiahs for sure. That is, the color of the ball would not affect your profits.*

Example 2: *If you choose to invest all of the 20,000 Rupiahs, then if the ball is blue, you win 60,000 Rupiahs and if the ball is red, you win nothing and end up with 0.*

Example 3: *If you choose to invest 10,000 Rupiahs, then if the ball is blue, you win 40,000*

*(20,000 − 10,000 +3 × 10,000), and if the ball is red, you win 10,000.*

### Genotyping

DNA has been collected from saliva on Indicating FTA-Elute paper (Millipore). A small disc was punched out and DNA eluted in 30 μl water. *DRD4* genotyping was adapted from Carpenter *et al*. (2011) with the following modification: 1 μl of DNA solution was amplified in 10 μl in a Roche LightCycler with 0,5 μM primers ggcacgtcgcgccaagctgca and ctgcgggtctgcggtggagtct, with the Qiagen multiplex PCR kit and the addition of Q solution and 100 μM dITP. An initial 15 sec denaturation at 95 °C to activate the enzyme, was followed by 35 cycles of 10” denaturation at 98 °C, 10” annealing at 68 °C and 2′ elongation at 72 °C. The PCR product was then run on a 2% agarose gel. Microsatellites were genotyped as described in^29^. D4S3248, D7S3056, D9S1121, D12S1300 and D13S894 were amplified with the Qiagen multiplex PCR kit, and analysed by capillary electrophoresis on a 3500xL Genetic Analyzer. Sequence of the primers:

D4S3248: 5′FAM ttcaggagtttagctttctatgc and ctacaccatcagtactcactaggc

D7S3056: 5′FAM caatagccctgaccttatgc and tacctacctacctacctctatggc

D9S1122: 5′AT550 gcttctgaaagcttctagtttacc and aatagtaatgccatttgtgatagg

D12S1300: 5′FAM cctcacaatgttgtaaggg and tgtaacatccgtgattaaaatagc

D13S894: 5′AT565 ggtgcttgctgtaaatataattg and cactacagcagattgcacca

### Statistics

In order to assess the importance of the minor alleles (other than 4 R and 2 R), we first built a model that includes the variables ‘Allele 2’ (number of copies of the allele 2 R), ‘Dominance’ (1 if the individual is heterozygote 4R-2R, 0 otherwise), ‘Allele x’ (1 if any allele other than 4 R or 2 R is present, 0 otherwise), ‘Area’, ‘Gender’, and ‘Income’ (standard score). Given that the variable ‘Allele x’ had a marginally significant effect (Wald test X² = 3.7, P = 0.055), the effect of minor alleles could not be neglected. We therefore searched for the best way to combine them with 4R and 2R. All possible groupings were generated, ‘Investment’ was fitted with a censored regression model (with the same control variables as above) for each grouping, and we relied on the AIC criterion to identify the best grouping method: alleles 3R and 6R with 2R, alleles 5R and 7R with 4R. Figs 2 and 3 are based on this grouping method. The results are similar when using only the individuals bearing only 4R or 2R alleles (Table S1, Figs S2 and S3).

### Population genetics

Each locus was tested for conformity with Hardy-Weinberg (HW) equilibrium using the exact U-score test with heterozygote deficiency being the alternative hypothesis^30^. A global test across samples and/or loci was also carried out^30^. Deviations from HW equilibrium were measured using the F_IS_ estimator^31^. Genotypic differentiation between populations was tested for by calculating an unbiased estimate of the *P*-value of a log-likelihood (G) based exact test^32^, a global test over loci was calculated using Fisher’s method. Population differentiation was measured using the F_ST_ estimator^31^. Calculations were performed using Genepop version 3.4^33^.

## Additional Information

**How to cite this article**: Faurie, C. *et al*. Evidence of genotypic adaptation to the exposure to volcanic risk at the dopamine receptor *DRD4* locus. *Sci. Rep.* **6**, 37745; doi: 10.1038/srep37745 (2016).

**Publisher's note:** Springer Nature remains neutral with regard to jurisdictional claims in published maps and institutional affiliations.

## Supplementary Material

Supplementary Information

## Figures and Tables

**Table 1:** Genotypic composition at the *DRD4* locus of populations living around the Merapi volcano, in risky and non risky areas.

Area	Genotypes											HW	
	2/2	2/4	3/4	4/4	2/5	4/5	2/6	4/6	2/7	4/7	All	F_IS_	*P*
Risky	23	44	—	21	—	1	0	1	1	3	94	0.003	0.519
Non-risky	25	61	1	64	4	3	3	2	0	1	164	0.111	0.181

Measures of departure from HW (Hardy-Weinberg) equilibrium (F_IS_) refer to the Weir and Cockerham’s estimate^31^. The *P*-value (*P*) corresponds to an exact test of departure from HW equilibrium with heterozygote deficiency as the alternative hypothesis. Genotype i/j refers to the *DRD4* genotype iR/jR.

**Table 2:** Genotypic differentiation between the risky and the non-risky areas.

Loci	F_ST_	*P*-value (SE)
*DRD4*	0.023	**0.010** (0.0007)
Microsatellites		
D4S3248	−0.0018	0.892 (0.0023)
D7S3056	0.0003	0.295 (0.0051)
D9S1122	−0.0002	0.413 (0.0056)
D12S1300	−0.0035	0.154 (0.0044)
D13S894	−0.0010	0.109 (0.0034)
All:	−0.0008	0.246

Measures of differentiation (F_ST_) refer to the Weir and Cockerham’s estimate^31^. *P*-values refer to an unbiased estimate of the *P*-value of a log-likelihood (G) based exact test^32^ and the global test over microsatellite loci was calculated using Fisher’s method. Significant *P*-values in bold characters.

**Table 3:** Censored regression model of investment.

a)					
	Estimate	SE	x^2^	df	*P*-value
Intercept	13,600	855			
Genotype			10.8	2	0.0046
*homo2/hetero*	1985	1231			
*homo4/hetero*	3116	1050			
Environment *(risky/non-risky)*	−1522	1009	2.3	1	0.13
Gender *(woman/ man)*	−4193	970	18.7	1	0.000015
Income	1826	511	12.8	1	0.00035
**b)**					
**Contrasts for the categories of the variable ‘genotype’.**			**x^2^**	**df**	***P*****-value**
*homo2/hetero*			5.9	1	0.015
*homo4/hetero*			8.8	1	0.003
*homo2/homo4*			0.011	1	0.92

Investment ranging between 0 and 20,000 IDR. N = 219 individuals (after excluding the missing data). a) For each variable, the estimate, standard error of the mean (S.E.), X^2^ statistic, degrees of freedom (df), and *P*-value of the Wald χ^2^ test are given. For categorical variables, the estimates are for one category compared to the reference category (underlined term). b) Contrasts for the three categories of the variable ‘genotype’ were performed. ‘*homo2*’ or ‘*homo4*’ refer to genotypes homozygote for allele 2 R or 4 R, respectively. ‘hetero’ refers to the genotype 2 R/4 R.
