# Corrigendum: Evidence of genotypic adaptation to the exposure to volcanic risk at the dopamine receptor *DRD4* locus

**DOI:** 10.1038/srep43978

**Published:** 2017-03-09

**Authors:** Charlotte Faurie, Clement Mettling, Mohamed Ali Bchir, Danang Sri Hadmoko, Carine Heitz, Evi Dwi Lestari, Michel Raymond, Marc Willinger

Scientific Reports 6: Article number: 3774510.1038/srep37745; published online: 12 01 2016; updated: 03 09 2017

The Acknowledgements section in this Article is incomplete.

“Genotyping Data used in this work were produced through the technical facilities of ISEM (Institut des Sciences de l’Evolution-Montpellier) and Labex Centre Méditerranéen Environnement Biodiversité. We thank L. Prezeau, E. Valjent and J. Shykoff for comments. MR was funded by grants from CNRS, MW by grants from IUF (Institut Universitaire de France) and Axa Research Foundation”.

should read

“Genotyping Data used in this work were produced through the technical facilities of ISEM (Institut des Sciences de l’Evolution-Montpellier) and Labex Centre Méditerranéen Environnement Biodiversité. We thank L. Prezeau, E. Valjent and J. Shykoff for comments. MR was funded by grants from CNRS, MW by grants from IUF (Institut Universitaire de France) and Axa Research Foundation. We are very grateful to Franck Lavigne, both for his personal involvement and for coordinating the SEDIMER project (sponsored by the AXA fund). We would also like to thank the students of UGM for their assistance for collecting the data and facilitating our field work”.

