# Supplementary material for: Evidence of genotypic adaptation to the exposure to volcanic risk at the dopamine receptor DRD4 locus
Source: Sci Rep. 2016 Dec 1;6:37745. doi: 10.1038/srep37745 (PMC5131341; doi:10.1038/srep37745)

Evidence of genotypic adaptation to the exposure to volcanic risk at the dopamine receptor DRD4 locus

Charlotte FAURIE[a,1], Clement METTLING[a,b,1], Mohamed Ali BCHIR[c], Danang Sri HADMOKO[d], Carine HEITZ[c], Evi Dwi LESTARI[d], Michel RAYMOND[a,2] and Marc WILLINGER[e,2]

a. Institute of Evolutionary Sciences (ISEM), University of Montpellier, 2 Place Eugène Bataillon, 34090 Montpellier, France.

b. Institute of Human Genetics, UPR 1142 CNRS, 141 rue de la Cardonille 34396 Montpellier, France

c. Ecole Nationale du Génie de l'Eau et de l'Environnement, 1 Quai Koch, 67070 Strasbourg, France

d. Universitas Gadjah Mada, Bulaksumur, Daerah Istimewa,Yogyakarta, Indonesia

e. Laboratoire Montpelliérain d'Economie Théorique et Appliquée, University of Montpellier, Bâtiment 26, 2 Place Pierre Viala, 34060 Montpellier, France

1 These authors contributed equally (co-first authors)

2 These authors contributed equally (co-last authors)

Correspondence to:

Michel Raymond: Institute of Evolutionary Sciences (ISEM), University of Montpellier, 2 Place Eugène Bataillon, 34090 Montpellier, France.

Tel: +33 4 67144615 email: michel.raymond@univ-montp2.fr

Marc Willinger: Laboratoire Montpelliérain d'Economie Théorique et Appliquée, University of Montpellier, Bâtiment 26, 2 Place Pierre Viala, 34060 Montpellier, France
Tel : +33 4 34432519 email : marc.willinger@lameta.univ-montp1.fr

Table S1. Censored regression model of investment excluding the individuals with minority alleles.

	**Estimate**	**SE**	**x^2**	**df**	***P*-value**
Intercept	14,140	918			
Genotype			8.8	2	0.012
homo2/hetero	2733	1280			
homo4/hetero	3012	1113			
Environment *(risky/non-risky)*	-1739	1067	2.7	1	0.10
Gender *(woman/ man)*	-4595	1015	20.5	1	0.00001
Income	1751	536	10.7	1	0.001

**Contrasts for the categories of the variable 'genotype'.**	**x^2**	**df**	***P*-value**
homo2/hetero	7.3	1	0.007
homo4/hetero	4.6	1	0.033
homo2/homo4	0.043	1	0.84

Investment ranged between 0 and 20,000 IDR. The individuals bearing minority alleles were excluded from the sample. For each variable, the estimate, standard error of the mean (S.E.), X^2 statistic, degrees of freedom (df), and *P*-value of the Wald χ2 test are given. For categorical variables, the estimates are for one category compared to the reference category (underlined term). Contrasts for the three categories of the variable 'genotype' were performed. N=219 individuals (after excluding the missing data).

Figure S1. Proportion of individuals investing each possible amount in the portfolio choice task. Individuals could choose to invest any amount between 0 and 20,000 in the risky option, by units of 500 IDR. Note that several amounts were never chosen (0, 500, 1500…). Individuals in the risky area (in grey) tend to be more risk-averse i.e. to invest less (on average 10,723 ± 529 SEM) than in the non-risky area (in black, 12,241 ± 450).

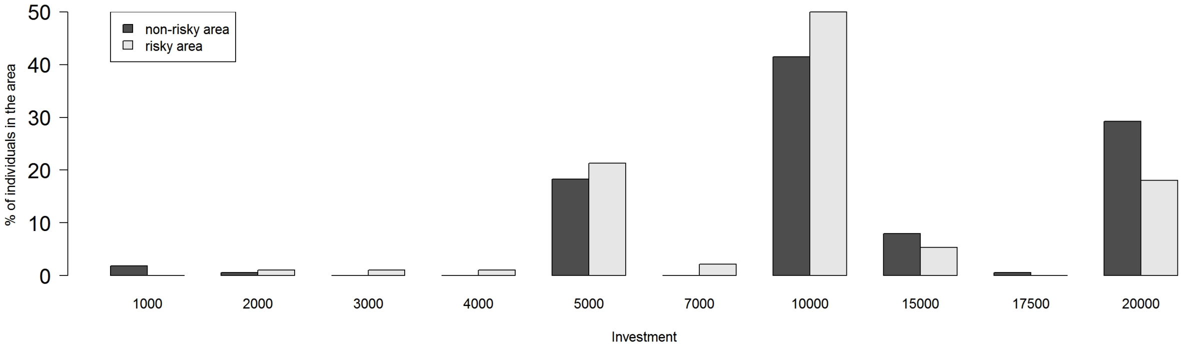

Figure S2. Average investment (in IDR) in the risky option, excluding the individuals with minority alleles. Heterozygotes 4R-2R tend to be risk-averse, i.e. to invest less money (11,300 ± 535) than homozygotes 4R-4R and 2R-2R (12,810 ± 636 and 12,467 ± 935, respectively). Sample sizes: 85 individuals with the genotype 4R-4R, 105 with 4R-2R, and 48 with 2R-2R.

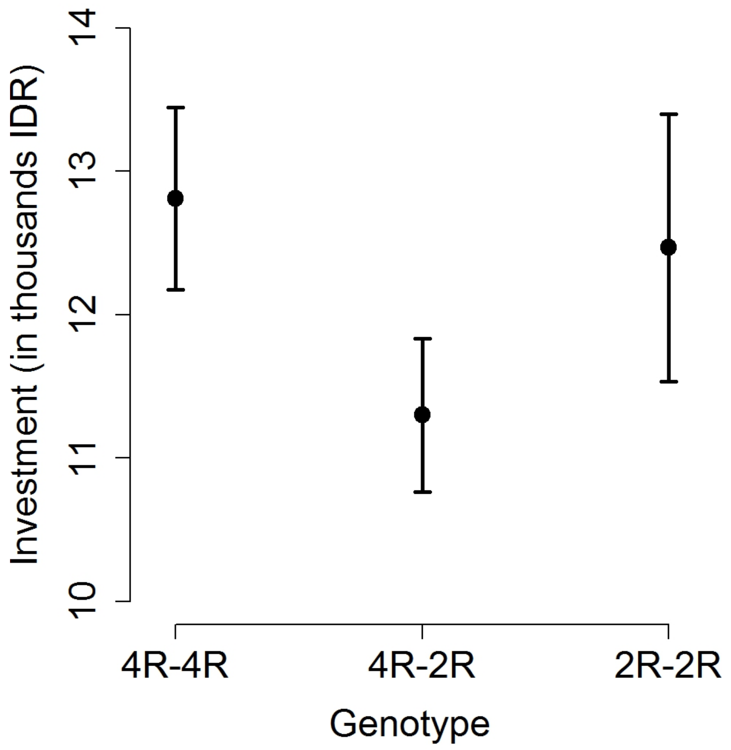

Figure S3. Proportion of individuals with the three possible genotypes, excluding the individuals with minority alleles. Non-risky area in black and risky area in grey. Heterozygotes 4R-2R are over-represented in the risky area. Sample sizes: 85 individuals with the genotype 4R-4R, 105 with 4R-2R, and 48 with 2R-2R.

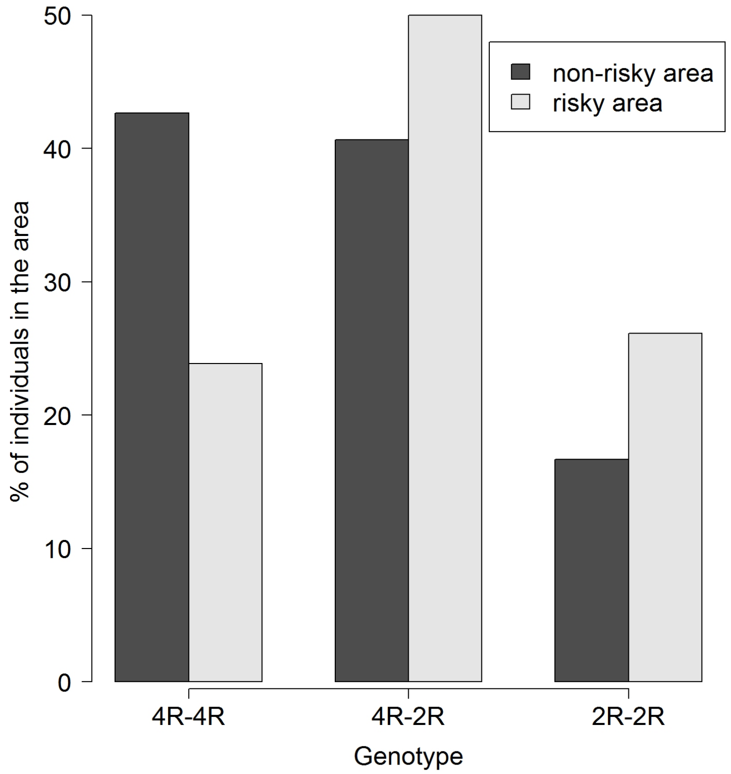

Supplement: Supplementary Information [file srep37745-s1.pdf]
